# Reference intervals for hemoglobin A1c (HbA1c) in healthy Mexican pregnant women: a cross-sectional study

**DOI:** 10.1186/s12884-018-2057-x

**Published:** 2018-10-29

**Authors:** Cristina M. Sánchez-González, Alfredo Castillo-Mora, Itzel N. Alvarado-Maldonado, Carlos Ortega-González, Nayeli Martínez-Cruz, Lidia Arce-Sánchez, Mabel Ramos-Valencia, Anayansi Molina-Hernández, Guadalupe Estrada-Gutierrez, Salvador Espino Y. Sosa, Yesenia Recio-López, Ruth Hernández-Sánchez, Enrique Reyes-Muñoz

**Affiliations:** 1 Division of Reproductive Medicine, Instituto Nacional de Perinatología Isidro Espinosa de los Reyes, Mexico City, Mexico; 2 Department of Endocrinology, Instituto Nacional de Perinatología Isidro Espinosa de los Reyes, Mexico City, Mexico; 3 Departament of Physiology and Cellular Development, Instituto Nacional de Perinatología Isidro Espinosa de los Reyes, Mexico City, Mexico; 4 Direction of Research, Instituto Nacional de Perinatología Isidro Espinosa de los Reyes, Mexico City, Mexico; 5 Division of Clinical Research, Instituto Nacional de Perinatología Isidro Espinosa de los Reyes, Mexico City, Mexico; 6 Division of Obstetrics and Gynecology, Hospital Regional Universitario de Colima, Colima, Mexico; 7 Department of Gynecological and Perinatal Endocrinology, Instituto Nacional de Perinatología Isidro Espinosa de los Reyes, Montes Urales 800, Lomas Virreyes, Miguel Hidalgo, CP 11000 Mexico City, DF Mexico; 8 Programa de Maestría en Ciencias Médicas de la Universidad Anáhuac Norte, Mexico City, Mexico

**Keywords:** Glycated hemoglobin, HbA1c, Pregnancy, Healthy pregnancy, Reference intervals, Gestational diabetes

## Abstract

**Background:**

The reference intervals for hemoglobin A1c (HbA1c) in pregnant Mexican women without diabetes are not well defined. The study aims to determine the reference intervals for HbA1c at each trimester in healthy Mexican pregnant women.

**Methods:**

This cross-sectional study included healthy Mexican pregnant women in trimester 1 (T1), 6–13.6 weeks of gestation (WG), trimester 2 (T2), 14–27 WG, and trimester 3 (T3), ≥27–36 WG, with a maternal age > 18 years, and pregestational body mass index (BMI) ranging between 18.5–24.9 kg/m^2^. Women with gestational diabetes mellitus, pregestational diabetes, anemia, a pregestational BMI < 18.5 or ≥ 25 kg/m^2^, and any hematologic, hepatic, immunological, renal, or cardiac disease were excluded. HbA1c was measured using high-performance liquid chromatography based on the National Glycohemoglobin Standardization Program-certified PDQ Primus guidelines. The HbA1c reference intervals were calculated in terms of the 2.5th to the 97.5th percentiles.

**Results:**

We analyzed the HbA1c values of 725 women (T1 *n* = 84, T2 *n* = 448, and T3 *n* = 193). The characteristics of the participants were expressed as mean ± standard deviation and included: maternal age (28.2 ± 6.7 years), pregestational weight (54.8 ± 5.9 Kg), pregestational BMI (22.2 ± 1.7 Kg/m^2^), and glucose values using a 75 g-2 h oral glucose tolerance test; fasting 4.5 ± 0.3 mmol/L (81.5 ± 5.5 mg/dL), 1 h 6.4 ± 1.5 mmol/L (115.3 ± 26.6 mg/dL), and 2 h 5.7 ± 1.1 mmol/L (103.5 ± 19.6 mg/dL). Reference intervals for HbA1c, expressed as median and 2.5th to 97.5th percentile for each trimester were: T1: 5.1 (4.5–5.6%), T2: 5.0 (4.4–5.5%), and T3: 5.1 (4.5–5.6%).

**Conclusions:**

The reference range of HbA1C in healthy Mexican pregnant women during pregnancy was 4.4% to 5.6%. We suggest as upper limits of HbA1c value ≤5.6%, 5.5%, and 5.7% for T1, T2, and T3, respectively among Mexican pregnant women.

## Background

Pregestational diabetes refers to any type of diabetes diagnosed before a pregnancy. Gestational diabetes mellitus (GDM) refers to diabetes diagnosed in the second trimester (T2) or third trimester (T3) of pregnancy that is not clearly overt diabetes [1]. The International Diabetes Federation estimated a global prevalence of 16.9% for hyperglycemia in pregnancy in 2013 [2].

During pregnancy, diabetes increases the risk of adverse perinatal outcomes, such as congenital malformations, macrosomia, preeclampsia, large fetus for gestational age, cesarean birth, and neonatal morbidity [3, 4]. The Hyperglycemia and Adverse Pregnancy Outcomes (HAPO) study reported associations between maternal glucose levels and increased birth weight, cesarean rate, and increased serum levels of C-peptide in the umbilical cord [5]. Several studies have shown that tight control of blood glucose levels during pregnancy may decrease the risk of adverse perinatal outcomes [6–8]. Good glycemic control is the first target of treatment for women with GDM [4, 8, 9].

According to the American Diabetes Association (ADA), health care providers and patients can use two techniques to evaluate the efficacy of glycemic control treatment: blood glucose self-monitoring (BGS) and hemoglobin A1c (HbA1c) [9]. An HbA1c target value ranging between 6 and 6.5% (42–48 mmol/mol) is recommended; however, an HbA1c of 6% (42 mmol/mol) may be optimal as a woman’s pregnancy progresses [9].

Some physiological changes in HbA1c during pregnancy should be considered to determine its optimal value for glycemic control. Erythrocytes half-life decreases during pregnancy, which is reflected in a decrease in HbA1c [10]. In addition, it has been shown that red cell turnover increases in a normal pregnancy, which contributes to a decrease in HbA1c [11]. These assertions suggest that, in order to ensure optimal glycemic control in pregnant woman with diabetes, it is necessary to use HbA1c reference values ​​specific for each trimester [9–11]. The decrease in HbA1c levels in the first trimester (T1) is known to be caused by lower pre- and postprandial mean blood glucose values ​​and an increase in young erythrocytes, which causes a decrease in the percentage of HbA1c [10]. The increase in HbA1c in T3 is caused by an increase in the mean postprandial blood glucose values [12].

Recent evidence has also shown that, despite optimal preconception control and unplanned pregnancies with good glycemic control in early pregnancy with optimal HbA1c levels, the development of complications associated with diabetes cannot always be prevented [13, 14].

These considerations highlight the need to carefully review glycemic control goals during pregnancy. Although HbA1c reference intervals for the general population are well established, they are not clearly defined in Mexican pregnant women. Therefore, the present study aimed to determine the reference intervals of HbA1c in each trimester of pregnancy in healthy Mexican pregnant women.

## Methods

### Design and participants

This cross-sectional study was approved by the Ethic and Research Internal Review Board of the Instituto Nacional de Perinatología in Mexico City, Mexico (register number 212250–42081). All the participants provided written informed consent. We included pregnant women who receive prenatal care at our institution from January 1, 2010 to April 30, 2011. Each of the women had a single pregnancy. Their maternal age was > 18 years old, and their pregestational body mass index (BMI) ranged between 18.5 kg/m^2^and 24.9 kg/m^2^. We excluded women with gestational diabetes mellitus (GDM) diagnosed using a 75 g-2 h oral glucose tolerance test (OGTT) with one or more of the following glucose cut-off points: fasting ≥5.1 mmol/L (92 mg/dL), 1 h ≥ 10 mmol/L (180 mg/dL), and 2 h ≥ 8.5 mmol/L (153 mg/dL) according to ADA criteria [1]. Women with pregestational diabetes mellitus diagnosed using a 75 g-2 h OGTT, defined by fasting ≥7 mmol/L (126 mg/dL) or 2 h ≥11.1 mmol/L (200 mg/dL), women with a pregestational BMI < 18.5 kg/m^2^ or ≥ 25 kg/m^2^, women with anemia defined by total hemoglobin concentration < 11 g/dL according to World Health Organization criteria [15], a multiple pregnancy, or any hematological, hepatic, immunological, renal, or cardiac disease were also excluded. Participants were divided into three groups based on the trimester (T) of pregnancy: T1: 6–13.6 weeks of gestation (WG), T2: 14–27.6 WG, and T3: 28–36 WG. Each participant fasted for 8 to 12 h prior to perform a 75 g-2 h OGTT as part of the universal 1-step screening method for GDM at first prenatal visit. Blood samples for measuring HbA1c level and fasting of OGTT were taken at the same time.

### Study variables

HbA1c was determined in plasma based on the National Glycohemoglobin Standardization Program–certified PDQ Primus guidelines (Primus Diagnostics, Kansas City, MO, USA) using high performance liquid chromatography (inter-assay CVs < 2%).

Glucose was measured using the Vitros DT60 II chemistry system (OrthoClinical Diagnostics, Tilburg, The Netherlands), according to the manufacturer’s instructions. The system has a sensitivity of 20 mg/dL (1.11 mmol/L) and a coefficient of variation of 1.4–1.8%.

The pregestational BMI was self-reported by each of the participants when the OGTT was conducted, and it was calculated using the following formula: weight in Kg /height in m^2^.

### Sample size

According to the International Federation of Clinical Chemistry (IFCC) [16, 17] recommendation on estimation of reference intervals the sample size must consist of a minimum of 40 participants in each group. Thus, we decided to include all the participants that met the study’s inclusion criteria.

### Statistical analysis

Descriptive statistics were used to characterize the three groups. The central tendency and/or frequency and percentage were measured, based on the type and distribution of each variable. An ANOVA test with Bonferroni correction was performed to compare the quantitative variables in each trimester. To determine the reference intervals, the median and 2.5th to 97.5th percentile were calculated according to the recommendations of the IFCC [16]. Statistical analysis was performed with the Statistical Package for the Social Sciences for Windows version 15.

## Results

**Fig. 1:**
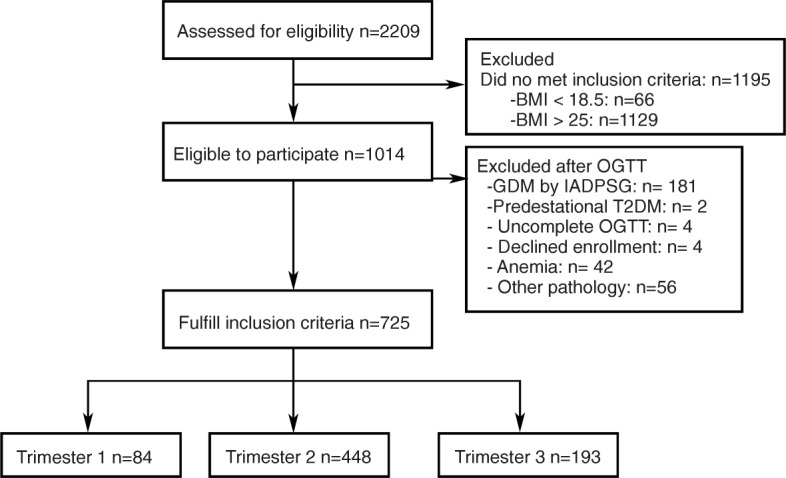
Flow chart of the study participants

During the study period, there were 2209 women assessed for eligibility at the study institution, 1014 of which were eligible to participate. Of these women, 725 met the inclusion criteria T1 (*n* = 84), T2 (*n* = 448), and T3 (*n* = 193); 289 were excluded because of GDM (*n* = 181), pregestational diabetes (*n* = 2), uncomplete OGTT (*n* = 4), declined enrollment (*n* = 4), anemia (*n* = 42), or some additional pathology (*n* = 56). Figure 1 shows the flow chart of the study participants.

**Fig. 2:**
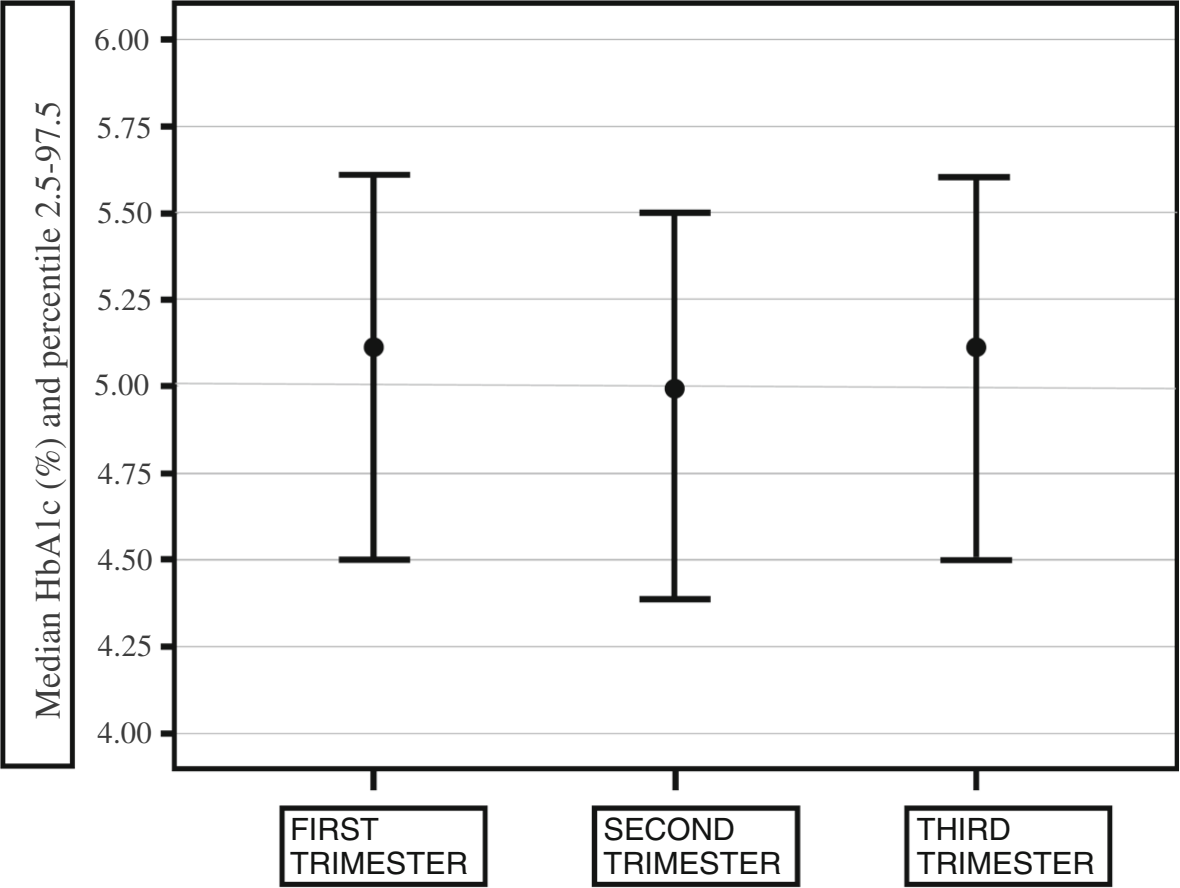
Median and percentile (2.5th to 97.5th) for HbA1c for Mexican women for each trimester

Figure 2 shows the reference intervals for HbA1c for T1 expressed as median and percentile (2.5th to 97.5th), which were: T1: 5.1% (4.5–5.6%), T2: 5.0% (4.4–5.5%) T3: 5.1% (4.5–5.6%). A statistically significant decrease was found between the T1 and T2 groups (*p* = 0.0001), and a statistically significant increase was found between the T2 and T3 groups (*p* = 0.0001). No statistically significant differences were found between the T1 and T3 groups.

The general characteristics of the 725 participants were expressed as mean ± standard deviation and included: maternal age (28.2 ± 6.7 years), pregestational weight (54.8 ± 5.9 Kg), pregestational BMI (22.2 ± 1.7 Kg/m^2^), maternal hemoglobin (13.2 ± 1.0 g/dL), and glucose values using a 75 g-2 h oral glucose tolerance test; fasting 4.5 ± 0.3 mmol/L (81.5 ± 5.5 mg/dL), 1 h 6.4 ± 1.5 mmol/L (115.3 ± 26.6 mg/dL), and 2 h 5.7 ± 1.1 mmol/L (103.5 ± 19.6 mg/dL).

Table 1 shows the characteristics of the participants at admission to the study. The weeks gestation at determination of HbA1c were 12.3 ± 1.6, 20.1 ± 3.6 and 30.4 ± 2.4 for T1, T2 and T3 groups, respectively. Maternal age was significantly higher in the T1 group than the T2 and T3 groups; however, pregestational BMI was similar in all three groups. Maternal hemoglobin was significantly higher in the T1 group (13.7 ± 1.1 g/dL) than the T2 (13.2 ± 0.96) and T3 groups (13. ± 1.01 g/dL). No statistically significant difference in glucose values obtained from the 75 g-2 h OGTT were observed for the T1 group in comparison to the T2 and T3 groups. The glucose values in OGTT were significantly higher in the T3 group than the T2 group.

**Table 1:** Characteristics per trimester of pregnancy of 725 healthy pregnant Mexican women

Characteristics	Group T1 *n* = 84	Group T2 *n* = 448	*p T1 vs T2	Group T3 *n* = 193	*p T1 vs T3
Maternal age (years)	30.1 ± 5.9	28.1 ± 6.7	0.04	27.5 ± 7.0	0.01
Pregestacional BMI (Kg/m^2^)	22.4 ± 1.6	22.3 ± 1.7	0.98	22.1 ± 1.6	0.78
Hemoglobin (g/dL)	13.7 ± 1.1	13.2 ± 0.96	0.0001	13.0 ± 1.0	0.0001
Hemoglobin HbA1c (%)	5.12 ± 0.2	5.0 ± 0.2	0.0001	5.1 ± 0.2**	0.98
Glucose values in OGTT (mmol/L)					
Fasting	4.56 ± 0.3	4.52 ± 0.3	0.74	4.52 ± 0.31	0.78
1-h	6.51 ± 1.6	6.2 ± 1.43	0.23	6.82 ± 1.45**	0.28
2-h	5.76 ± 1.2	5.65 ± 1.05	0.98	6.0 ± 1.08 **	0.22

*ANOVA Test

**p < 0.0001 for the comparison T2 Vs T3

*BMI* Body mass index, *OGTT* Oral glucose tolerance test

## Discussion

In our study, we found that the HbA1c reference interval in healthy Mexican pregnant women in the 97.5th percentile for the T1, T2, and T3 groups was ≤5.6%, 5.5%, and 5.6%, respectively. Our findings are important because this is the first study to evaluate HbA1c levels in healthy Mexican pregnant women.

While HbA1c levels have been reported to be lower in healthy pregnant women in comparison to non-pregnant women [14], there is controversy regarding whether there are differences in HbA1c reference intervals at each trimester of pregnancy. Worth et al. [18] reported a significant increase among T1, T2, and T3 groups. Versantvoort et al. [19] reported a small decrease in HbA1c levels during T1 (5.4%), compared with T2 (5.5%), and T3 (5.8%). They also suggested a correlation between HbA1c levels during T1 and T2 and the birth weight percentile [19]. However, Hartland et al. [20], O’Kane et al. [21], Hanson et al. [22], and Günter et al. [23], Hiramatsu et al. [24] reported a significant decrease in HbA1c in T2, which is similar to our findings.

Evers et al. [25] conducted a nationwide study in the Netherlands on the risk of complications in pregnant women with type 1 diabetes mellitus (DM1). They reported that the incidence of all congenital malformations in women with HbA1c levels of 6.3% during T1 was twice that of the population without DM1. They also reported that the incidence of congenital malformations was 12.9% in women with HbA1c levels > 7%; they concluded that maintaining HbA1c < 7% does not decrease the risk of congenital malformations [25]. Another study on this same population reported that the incidence of macrosomia was very high (48%) despite the fact that 84% of women with DM1 had good glycemic control (HbA1c < 7%) [26].

Radder et al. [27] suggested that to prevent congenital malformations and macrosomia in diabetic and pregnant women, HbA1c levels should be < 5% in T1 and less than 6% in T3. Mosca et al. [28] reported a lower level of HbA1c in 445 Italian pregnant women (median 4.8% and the 2.5th to 97.5th percentile [4–5.5%]) in comparison to 384 control women without pregnancy (median 5.6 and the 2.5th to 97.5th percentile (4.8–6.2%). While Mosca et al. [28] did not report the BMI or the age of the women in their study, the reference interval for HbA1c was similar to the interval in our study.

Our study had several limitations. Due to the study’s design, HbA1c was determined for different women in each trimester. The pregestational BMI was self-reported by the participants, so it could be less exact than pregestational BMI that is documented by a clinician. The study results are only applicable to Mexican women and, potentially, Latin women. Future prospective and multi-center studies are needed to corroborate our findings.

Our study also has several strengths. It is the first study to evaluate HbA1c in healthy Mexican woman; pregestational and gestational diabetic women were excluded using OGTT, and the sample size included women in each trimester.

Diabetes in pregnancy involves an additional risk for both the mother and the fetus and it is directly related to glycemic control, which is evaluated using the HbA1C value. This correlation highlights the importance of accurate measurement as well as correct interpretation of and comparison with the appropriate reference values. HbA1c reference values ​​per trimester of pregnancy are necessary in order to ensure better management of women with pregnancies complicated by diabetes because strict glycemic control is essential in order to minimize maternal and fetal morbidity [26, 29]. Several authors have demonstrated that the measurement of HbA1c is a useful parameter in glycemic control [20, 21, 30]; Therefore, we suggest that these results be considered when determining treatment goals in Mexican women with diabetes during pregnancy, however studies among diabetic women using this reference value for HbA1c are needed.

## Conclusions

The HbA1C reference range for healthy Mexican pregnant women during pregnancy is 4.4% to 5.6%. Based on our results, we suggest as upper limits of HbA1c value ≤5.6%, 5.5%, and 5.6% for T1, T2, and T3, respectively among Mexican pregnant women.

## Abbreviations

ADA: American Diabetes Association
BMI: Body mass index
DM1: Diabetes Mellitus Type 1
DM2: Diabetes Mellitus Type 2
GDM: Gestational Diabetes Mellitus
HbA1C: Glycated hemoglobin
WG: Weeks of gestation
